# Unanchored simulated treatment comparison on survival outcomes using parametric and Royston-Parmar models with application to lenvatinib plus pembrolizumab in renal cell carcinoma

**DOI:** 10.1186/s12874-025-02480-x

**Published:** 2025-01-30

**Authors:** Christopher G. Fawsitt, Janice Pan, Philip Orishaba, Christopher H. Jackson, Howard Thom

**Affiliations:** 1 Clifton Insight, Bristol, UK; 2 Eisai, Nutley, NJ 07110 USA; 3 MRC Biostatistics Unit, University of Cambridge, Cambridge, UK; 4 University of Bristol, 1-5 Whiteladies Rd, Clifton, UK

**Keywords:** Simulated treatment comparison, Royston-Parmar spline models, Parametric models, Population-adjusted methods, Overall survival, Progression-free survival, Renal cell carcinoma

## Abstract

**Background:**

Population-adjusted indirect comparison using parametric Simulated Treatment Comparison (STC) has had limited application to survival outcomes in unanchored settings. Matching-Adjusted Indirect Comparison (MAIC) is commonly used but does not account for violation of proportional hazards or enable extrapolations of survival. We developed and applied a novel methodology for STC in unanchored settings. We compared overall survival (OS) and progression-free survival (PFS) of lenvatinib plus pembrolizumab (LEN + PEM) against nivolumab plus ipilimumab (NIVO + IPI), pembrolizumab plus axitinib (PEM + AXI), avelumab plus axitinib (AVE + AXI), and nivolumab plus cabozontanib (NIVO + CABO) in patients with advanced renal cell carcinoma (RCC). Unanchored comparison was necessitated as the control groups differed in their use of PD-1/PD-L1 rescue therapy.

**Methods:**

We fit covariate-adjusted survival models to individual patient data from phase 3 trial of LEN + PEM, including standard parametric distributions and Royston-Parmar spline models with up to 3 knots. We used these models to predict OS and PFS in the population of comparator treatments. The base case model was selected by minimum Akaike Information Criterion (AIC). Treatment effects were measured using difference in restricted mean survival time (RMST), over shortest follow-up of input trials, and hazard ratios at 6, 12, 18, and 24 months.

**Results:**

The survival model with the lowest AIC was 1-knot spline odds for OS and log-logistic for PFS. Difference in RMST OS was 6.90 months (95% CI: 1.95, 11.36), 5.31 (3.58, 7.28), 5.99 (1.82, 9.42), and 11.59 (8.41, 15.38) versus NIVO + IPI (over 64.8 months follow-up), AVE + AXI (46.7 months), PEM + AXI (64.8 months), NIVO + CABO (53.0 months), respectively. Difference in RMST PFS was 4.50 months (95% CI: 0.92, 8.26), 8.23 (5.60, 10.57), 5.38 (2.06, 9.09), and 4.58 (0.09, 9.44) versus NIVO + IPI (over 57.8 months), AVE + AXI (44.9 months), PEM + AXI (57.8 months), NIVO + CABO (23.8 months), respectively. Hazard ratios indicated strong evidence of greater OS and PFS on LEN + PEM at most timepoints.

**Conclusions:**

We developed and applied a novel methodology for comparing survival outcomes in unanchored settings using STC. Pending investigation with a simulation study or further examples, this methodology could be used for clinical decision-making and, if long-term data are available, inform economic models designed to extrapolate outcomes for the evaluation of lifetime cost-effectiveness.

**Trial registration:**

NCT02811861 (registered: 23/06/2016).

**Supplementary Information:**

The online version contains supplementary material available at 10.1186/s12874-025-02480-x.

## Background

Cancer treatments designed to improve patients’ length of life are ideally evaluated in clinical trials, where the key outcomes, or endpoints, used to establish efficacy often include overall survival (OS) and progression-free survival (PFS); the length of time after receiving a treatment for which the disease does not progress and patients do not die. Comparative assessment of survival outcomes across new and existing treatments is crucial to guide clinical decision making, as well as health technology assessment (HTA) and economic evaluation, which many international regulatory agencies rely on to determine the most cost-effective use of resources [1–3]. In the absence of direct head-to-head evidence on survival outcomes, indirect treatment comparison (ITC) methods, such as network meta-analysis (NMA), can be used to assess relative efficacy across interventions [4, 5]. However, NMA relies on a shared comparator to connect treatments within an evidence network, which may not always exist; for example, trials may lack a control arm (e.g., single-arm trials), or the control arm may differ substantially across trials, leading to a disconnected network [6]. Such a comparison is unanchored, rather than anchored by a common control, and it is not feasible to use NMA in this setting.

Unanchored population-adjusted indirect treatment comparisons are increasingly used in submissions to reimbursement agencies, such as the National Institute for Health and Care Excellence (NICE) [6, 7]. These methods include matching-adjusted indirect comparison (MAIC), simulated treatment comparison (STC), and more recently, multi-level network meta-regression (ML-NMR) [6, 8, 9]. Population-adjusted methods take advantage of individual patient data (IPD) for at least one comparator and use evidence on potential effect modifying variables and prognostic factors when establishing estimates of relative efficacy in another population.

Comparative assessment of survival outcomes for disconnected networks has almost exclusively relied on MAIC in the literature [10]. This approach utilises IPD for the intervention of interest and re-weights patients’ so that their weighted average baseline characteristics match those of the comparator population (for which only aggregate data are available) [9]. The weights are typically obtained using propensity scores, estimated with method of moments, but can also be obtained using entropy balancing [9, 11]. In both cases, weighted Cox analysis is used to generate hazard ratios between the intervention of interest and the comparator, based on aggregate data for comparator treatments, including digitized Kaplan–Meier (KM) survival curves [12]. Intervention patient data are weighted by their estimated weights while equal weights are applied to the comparator patient data. A problem with the Cox model is that it relies on the assumption of proportional hazards (i.e., it assumes the relative hazard remains constant over time). This is likely an over-simplification for many survival outcomes for which hazard ratios are typically expected to vary over time, such as in melanoma, non-small cell lung cancer and renal cell carcinoma (RCC) [13–15]. One further issue related to HTAs and economic models in general is that MAIC provides no extrapolation options to project survival outcomes beyond observed trial data. A MAIC hazard ratio can be applied to a survival model for the comparator treatment but this relies on proportional hazards being valid over the whole extrapolation period, and requires a separate survival model to be fit [16, 17]. This is a major limitation of MAIC in the context of HTA submissions, for example, where extrapolations are necessary to evaluate lifetime costs and consequences [18].

STC provides an alternative population-adjusted indirect comparison method in unanchored settings. STC fits parametric models and provides extrapolation options for use in HTAs and economic models. However, methodological exploration of the application of STC to survival outcomes has been limited [19]. STC uses multivariable regression (on all potential effect modifiers and prognostic variables) to predict the event time curve (e.g., OS) at the baseline characteristics of comparator trials. This prediction is compared to the KM curves of the unadjusted data for the comparator treatment to produce an estimate of relative efficacy [7]. As with MAIC, the target population is that of the comparator treatment rather than the intervention of interest [6]. A shortcoming of both STC and MAIC is that they may be biased if they have not included all important effect modifiers or prognostic variables [6]. However, simulation studies in the anchored setting have found that STC gives less biased estimates than MAIC [20]. Simulation studies have also shown that MAIC is sensitive to model misspecification [21].

In anchored settings (i.e., standard NMA), survival outcomes are typically evaluated using standard parametric survival curves (e.g., Weibull or Gamma distributions), fractional polynomials, and/or Royston Parmar spline models [22–24]. Spline models are now commonly applied in oncology settings and have been found to outperform standard parametric models by better representing the early complexity of hazard functions, as well as declining hazards in the tails of the functions if such a decline is clinically plausible or supported by data [25]. Spline models are also recommended as options for extrapolation in economic evaluation and HTA although they need to be guided by long-term data [18, 26, 27]. Spline NMA can give better fit to data and, in cases where external data are available, more sensible extrapolation than NMA based on fractional polynomials since they’re forced to be linear at the end of the curve, thereby reducing the possibility of unexpected end-effects [13]. While spline NMA relies on connected networks, covariate-adjusted Royston Parmar spline models can be applied to unanchored STC [23].

In the absence of a specific methodology for evaluating survival outcomes in unanchored settings using STC, we developed and applied a novel approach that fits both standard parametric and Royston-Parmar spline models to survival data, which avoids the assumption of proportional hazards. Our analysis focussed on survival outcomes in advanced RCC using evidence from a phase 3 trial which compared lenvatinib plus pembrolizumab (LEN + PEM) with sunitinib (SUN) as first-line therapy, along with published evidence on recent immunotherapy treatments.

### Application to lenvatinib plus pembrolizumab for renal cell carcinoma

Characterised by susceptibility to both immunotherapeutic and antiangiogenic treatment approaches and resistance to cytotoxic chemotherapy, RCC accounts for 2% of global cancer diagnoses and deaths [28, 29]. In the United States alone, RCC is responsible for more than 14,400 deaths annually [30]. Metastatic RCC has only a 12% 5-year survival rate [28]. Until recently, treatments that target the vascular endothelial growth factor (VEGF) pathway, such as SUN, had been standard first-line therapy for advanced disease [30]. Standard-of-care now consists of immune-checkpoint inhibitors, either as dual-type combination (e.g., nivolumab plus ipilimumab [NIVO + IPI]) or combined with kinase inhibitors (e.g., pembrolizumab plus axitinib [PEM + AXI], avelumab plus axitinib [AVE + AXI], nivolumab plus cabozontanib [NIVO + CABO]), which have been shown to have better outcomes than SUN [31–34].

LEN is an oral small-molecule inhibitor of receptor tyrosine kinases (RTKs) that are overexpressed in many cancers [35]. In combination with PEM and platinum doublet chemotherapy (chemotherapy), a Phase 1b-2 RCT found promising tumour response to lenvatinib plus pembrolizumab (LEN + PEM) in previously treated patients with RCC. In a follow-up multicentre, open-label, randomized Phase 3 trial (CLEAR), LEN + PEM was shown to significantly improve OS and PFS against SUN as first-line therapy in advanced RCC [36].

NMA has been used to examine the relative effects of LEN + PEM versus comparator first-line treatments in advanced RCC, including NIVO + IPI, AVE + AXI, PEM + AXI, and NIVO + CABO, which connected via SUN [37]. However, the anchored treatment comparison was likely biased since the control arm (SUN) in CLEAR differed substantially from the control arm (SUN) in comparator trials due to use of differing rescue therapies. In CLEAR, the proportion of patients that discontinued treatment and received subsequent systemic therapy, including anti-programmed death-1 (PD-1) and programmed death-ligand 1 (PD-L1) inhibitors, was higher than most comparator trials [37]. At latest follow-up, 68.9% of SUN-treated patients that discontinued treatment received subsequent systemic therapy in CLEAR, of which 54.6% received PD-1 or PD-L1 inhibitor [32]. In contrast, 41.0% of SUN-treated patients in CheckMate 9ER, which compared NIVO + CABO with SUN, received subsequent anti-cancer therapy, of which 31.0% received PD-1 or PD-L1 inhibitor [31]. Similarly low proportions of patients received subsequent systemic therapy, including PD-1 or PD-L1 inhibitor, in CheckMate 214 and JAVELIN RENAL (Table 1) [33, 38]. In contrast, 73.6% of SUN-treated patients in KEYNOTE-426 received subsequent anti-cancer therapy, of which 80.0% received PD-1 or PD-L1 inhibitor [34]. As a consequence, the control group in CLEAR (and KEYNOTE-426) likely have a greater survival advantage relative to control groups in comparator trials; therefore, any comparison of relative effects among treatments anchored on SUN would bias against LEN + PEM. Unanchored population adjusted indirect comparison methods are therefore needed for unbiased comparison.

**Table 1:** Summary of rescue therapies in the control arms of included trials at latest follow up

Trial	Comparator	Control	% of control group receiving anti-cancer therapy	% that received anti-PD-1/PD-L1 inhibitor*
CheckMate 214 [33]	NIVO + IPI	SUN	61%	35%†
CheckMate 9ER [31]	NIVO + CABO	SUN	41%	31%
CLEAR	LEN + PEM	SUN	68.9%	54.6%
JAVELIN RENAL [38]	AVE + AXI	SUN	60.6%	37.2%
KEYNOTE-426 [34]	PEM + AXI	SUN	73.9%	80.0%

^*^Percentage of control group that received anti-cancer therapy

^†^Only reported most common PD-1 inhibitor: nivolumab

*AVE + AXI *avelumab + axitinib, *LEN + PEM* lenvatinib + pembrolizumab, *NIVO + CABO* nivolumab + cabozantinib, *PD-L1* programmed death ligand 1, *PEM + AXI* pembrolizumab + axitinib, *SUN* sunitinib, *AVE + AXI* avelumab + axitinib, *LEN + PEM* lenvatinib + pembrolizumab, *NIVO + CABO* nivolumab + cabozantinib, *PD-L1* programmed death ligand 1, *PEM + AXI* pembrolizumab + axitinib, *SUN* sunitinib

### Methods

Below we present the data used in our analysis, followed by the methods and parameterisation for STC parametric models and Royston Parmar spline models, and statistical analysis performed.

### Data

IPD for LEN + PEM were derived from CLEAR, a phase 3 trial that compared the efficacy (including OS and PFS) and safety of the combination therapy with SUN in patients with advanced RCC [36]. First-line treatment comparators included recent immunotherapy treatments for advanced RCC; these included the following treatments:

- PEM + AXI (KEYNOTE-246) [34]
- AVE + AXI (JAVELIN) [38]
- NIVO + IPI (CheckMate 214) [33]
- NIVO + CABO (CheckMate 9ER) [31]

Survival outcomes for comparator treatments derived from each trial using published KM curves, from which we reconstructed IPD using the Guyot method [39]. Common baseline characteristics that were reported across each of the trials are summarized in Table 2, and include age, gender, race, region, MSKCC-favourable risk (Memorial Sloan Kettering Cancer Center) favourable, IMDC-favourable risk (International Metastatic RCC Database Consortium) favourable, PD-L1 < 1, number of metastatic sites ≥ 2, and location of lesions (bone, lymph node, liver or lung). Both MSKCC-favourable risk and IMDC-favourable risk are informed by the prognostic models developed by Motzer and colleagues to classify patients into three risk groups based on the number of risk factors; these include favourable-, intermediate-, and poor-risk [40].

**Table 2:** Common baseline characteristics reported across trials included in the regression models for unanchored STC

Study	Comparator	Median age (min, max) (years)	Proportion female	Race: White	Region: Rest of the world	Proportion IMDC FAVORABLE	Proportion MSKCC FAVORABLE	Proportion with PD-L1 < 1%	Proportion with number of metastases ≥ 2	Proportion with bone lesions	Proportion with lymph node lesions	Proportion with liver lesions	Proportion with lung lesions
CLEAR	LEN + PEM	62 (34, 88)	0.28	0.736	0.440	0.31	0.29	0.315	0.715	0.22	0.45	0.18	0.71
CheckMate 214 [33]	NIVO + IPI	62 (26, 85)	0.25	NR	0.35	0.23	NR	0.77	0.78	0.2	0.45	0.18	0.69
CheckMate 9ER [31]	NIVO + CABO	62 (29, 90)	0.229	0.83	0.511	0.229	0.8	0.743	0.802	0.241	0.402	0.226	0.737
JAVELIN RENAL 101 [38]	AVE + AXI	62 (29, 83)	0.285	0.781	0.421	0.213	0.217	NR	0.566	NR	NR	NR	NR
KEYNOTE-426 [34]	PEM + AXI	62 (30, 89)	0.287	NR	0.514	0.319	NR	0.407	0.729	0.238	0.461	0.153	0.722

*AVE + AXI* avelumab + axitinib, *LEN + PEM* lenvatinib + pembrolizumab, *NIVO + CABO* nivolumab + cabozantinib, *PD-L1* programmed death ligand 1, *PEM + AXI* pembrolizumab + axitinib

### Simulated treatment comparison notation

Suppose we are comparing LEN + PEM (treatment 1) using the CLEAR trial (population 1) with PEM + AXI (treatment 2) using the KEYNOTE-426 trial (population 2). We use the notation ${\widehat{Y}}_{k\left(i\right)}$ for absolute response in treatment $k$ of study population $i$, with the index $i$ in parenthesis denoting the population. An unanchored indirect comparison would generate an estimate ${\widehat{Y}}_{1\left(2\right)}$ of absolute response, such as the OS or PFS survival curve, of treatment LEN + PEM in the population of the PEM + AXI arm of KEYNOTE-426. This is compared with the unadjusted outcome ${\widehat{Y}}_{2(2)}$, which in our setting is the KM curve for OS or PFS. The estimator of the relative effect between 1 and 2 and is

1 $${\widehat d}_{12(2)}=\mathrm g\left({\widehat Y}_{2(2)}\right)-\mathrm g\left({\widehat Y}_{1\left(2\right)}\right)$$

where the $\text{g}()$ is some link function transforming the outcome to a suitable scale for indirect comparisons, such as that of log hazard rates [6].

Unanchored indirect comparisons must adjust for imbalance in both effect modifiers and prognostic variables. Prognostic variables are those that affect absolute outcomes, regardless of treatment. Randomised trials balance prognostic variables in the treatment 1 and 2 arms so purely prognostic variables are not a concern for NMA. Effect modifiers are those that affect treatment effects.

In an unanchored comparison, the target population of comparison is that of treatment 2 (i.e., in the “comparator” trial). In our analysis, we used IPD from LEN + PEM (i.e., treatment 1) in CLEAR to construct an outcome regression model and predict response in the population of interest in comparator trials (i.e., NIVO + IPI, AVE + AXI, PEM + AXI, and NIVO + CABO).

The models for patient $j$ in the treatment 1 arm of IPD study $i$ used are accelerated failure time models (e.g., exponential, Weibull, log-logistic) with hazard function (for time to mortality for OS or time to progression or death for PFS)

2 $$\lambda_{ij}\left(t\vert\theta_{ij}\right)=\theta_{ij}\;\lambda_0\left(t\theta_{ij}\right)$$

where $t$ is time. The survivor function is

3 $${\mathrm S}_{ij}\left(t\vert\theta_{ij}\right)=S_0\left(t\theta_{ij}\right)$$

where ${\theta }_{ij}$ is the “time acceleration” factor, which depends on the patient’s covariate values through a log-linear model: [41]

4 $$\log\left(\theta_{ij}\right)=\mu+{\boldsymbol X}_{ij}\cdot\boldsymbol\beta$$

For example, doubling the value of a covariate with coefficient $\beta =\text{log}\left(2\right)$ will give half the expected survival time.

The parameter $\mu$ is an intercept, ${{\varvec{X}}}_{ij}$ is a vector of relevant patient characteristics (i.e., treatment effect modifiers or prognostic variables), and ${\varvec{\beta}}$ is a vector of regression coefficients.

Using the estimates $\widehat{\mu }$ and $\widehat{{\varvec{\beta}}}$ a population adjusted estimator $\text{log}\left({\widehat{\theta }}_{1\left(2\right)}\right)$ is formed using the mean covariate values ${\overline{{\varvec{X}}} }_{(2)}$ from the comparator arm (population 2). The final STC estimator for the outcome on treatment 1 in population 2 is then

5 $$\log\left({\widehat\theta}_{1\left(2\right)}\right)=\widehat\mu+{\overline{\boldsymbol X}}_{(2)}\cdot{\widehat{\boldsymbol\beta}}$$

The estimated hazard for a patient with average characteristics at all times $t$ can then be calculated as ${\widehat{\theta }}_{1\left(2\right)}\widehat{{\uplambda }_{0}}\left(t{\widehat{\theta }}_{1\left(2\right)}\right)$, where the baseline hazard ${\uplambda }_{0}$() depends on the selected distribution. Hence the survivor function for an average patient can be calculated at all times. We consider the standard parametric models implemented in the flexsurv package for R, namely exponential, Weibull, gamma, log-logistic, log-normal, and generalized F [42]. The Gompertz model is also considered, which is not an accelerated failure time parameterisation, but instead characterised as a proportional hazards model. The models fit to the LEN + PEM KM data are independent of the comparator KM data, so proportional hazards are not assumed for any models.

### Royston parmar spline models notation

Royston Parmar spline models have a different parametrisation [42]. If $S\left(t\right)$ is the survival function at time $t$ with log time $u=\text{ln}\left(t\right)$ a spline is defined as

6 $$g\left(S\left(t\right)\right)=s\left(u,\gamma\right)=\gamma_0+\gamma_1u+\gamma_2v_1\left(u\right)+\dots+\gamma_{m+1}v_m\left(u\right)$$

where $g()$ is a link function and estimated parameters are ${\varvec{\gamma}}$. Boundary knots ${k}_{min}$ and ${k}_{max}$ plus $m\ge 0$ internal knots are placed on the axis of log time and used to define the $m$ restricted cubic basis functions ${v}_{j}\left(u\right)$ as

$${v}_{j}\left(u\right)={\left(u-{k}_{j}\right)}_{+}^{3}-{\vartheta }_{j}{\left(u-{k}_{min}\right)}_{+}^{3}-\left(1 -{\vartheta }_{j}\right){\left(u-{k}_{max}\right)}_{+}^{3}$$

7 $$\vartheta_j=\frac{k_{max}-k_j}{k_{max}-k_{min}}$$

With ${\left(u-a\right)}_{+}=\text{max}\left(0, u-a\right)$. Covariates for STC regression are included on the ${\gamma }_{0}$ linear parameter, defining an extended model as

8 $$g\left(S\left(t,{\boldsymbol X}_{ij}\right)\right)=s\left(u,\gamma\right)+{\boldsymbol X}_{ij}\cdot\boldsymbol\beta$$

Various forms for the link function can be selected:

1. Log cumulative hazards, which defines an extension of the proportional hazards Weibull model:

9 $$g\left(S\left(t,\;{\boldsymbol X}_{ij}\right)\right)=\log\left(-\log\left(S\left(t,\;{\boldsymbol X}_{ij}\right)\right)\right)$$

2. Log cumulative odds, which defines an extension of the proportional odds log-logistic model:

10 $$g\left(S\left(t,\;{\boldsymbol X}_{ij}\right)\right)=\log\left({S\left(t,\;{\boldsymbol X}_{ij}\right)}^{-1}-1\right)$$

3. Inverse Normal, which defines an extension of the log-normal model:

11 $$g\left(S\left(t,\;{\boldsymbol X}_{ij}\right)\right)=\Phi^{-1}\left(S\left(t,\;{\boldsymbol X}_{ij}\right)\right)$$

The “proportional hazards” and “proportional odds” assumptions in these models are only applied to the effect of covariates other than treatment. In unanchored STC, a survival curve is fit to the LEN + PEM arm and KM data used for the comparator, so proportional hazards or proportional odds are not assumed.

Once the model is fit to the LEN + PEM arm of CLEAR, it can be used to predict a survival curve at the average characteristics of comparator arms ${\overline{\boldsymbol X}}_{(2)}$ for each time $t$

12 $$g\left(S\left(t,{\overline{{\varvec{X}}} }_{(2)}\right)\right)=s\left(u,\widehat{\gamma }\right)+{\overline{{\varvec{X}}} }_{(2)}\bullet \widehat{{\varvec{\beta}}}$$

#### Statistical analysis

Model selection was decided on the basis of lowest AIC of model fit to the LEN + PEM arm of CLEAR; if the models had been used for extrapolations, long-term external data would be needed to validate their clinical plausibility [18, 43–45]. Models for which the parameter estimation procedure did not converge were not considered. We considered 16 possible survival distributions. These included the 7 standard parametric (i.e., exponential, Weibull, Gompertz, gamma, log-logistic, log-normal, generalised-F) plus 9 spline models with up to 3 knots using hazard, odds and normal links.

In line with published recommendations and NICE guidance, we considered all possible effect modifiers and prognostic factors [6, 46]. There were 10 possible covariates to include, which were those recorded by CLEAR and reported by at least one of the comparator studies. Since the number of potential models that could be fitted was very large, we assumed that the optimal selection of covariates did not depend on the baseline survival distribution. Hence, we first explored the best survival distribution with no covariates (16 possible models), choosing lowest AIC distribution (plus next three for sensitivities). We then chose a covariate combination (from 1,024 possibilities) for the distribution with lowest AIC.

We considered 6 sensitivity analyses in total. These were the 3 lowest AIC survival distributions with the final selection of covariates; the lowest AIC survival distribution with all and no covariates; and a model with no adjustment of CLEAR (i.e., using raw KM data). If a selected covariate was not reported by a comparator study, it was not included in the regression model; the result is that different models can be used for each comparison.

To summarise differences between treatments, we used the hazard ratio at 6, 12, 18, and 24 months and difference in restricted mean survival time (RMST). Hazard ratios are calculated on the log scale using the difference in log hazards between the two treatments; these vary with time, thus giving non-proportional hazards, for all models except the exponential. As no parametric model is fit to the comparator data, a kernel density of the log hazard is estimated from the KM data using the muhaz package of R [47, 48]. The RMST was calculated up to the CLEAR horizon of 64.8 months for OS and 57.8 months for PFS, or the time horizon of the comparator trial, depending on which was shortest. RMST on the LEN + PEM arm of CLEAR was calculated as the area under the predicted survival curve. RMST was used for the comparator arm on which only KM data were available [49]. In the sensitivity analysis where only KM data were used for CLEAR, restricted mean survival was also calculated for LEN + PEM. Uncertainty was represented by 1,000 bootstrap resamples of patients from CLEAR and survival times from KM data. Medians and 95% confidence intervals are reported for difference in mean survival and hazard ratio.

All analyses were coded in the R statistical programming language using the ‘flexsurv’ package [42, 50].

## Results

### OS

The AIC of the various survival models fit to OS are summarised in Additional File 1, along with the 10 covariate-adjusted (1-knot spline odds) models with lowest AIC. Overall, there was little difference in AIC between the models, although the survival model with the lowest AIC was a 1-knot spline odds. The base case model included all prognostic factors and treatment effect modifiers, in line with NICE guidance [7]; this led to a regression model that adjusted for age, sex, MSKCC, IMDC, number of metastatic sites and all lesion locations (bone, lymph, liver, lung).

**Fig. 1:**
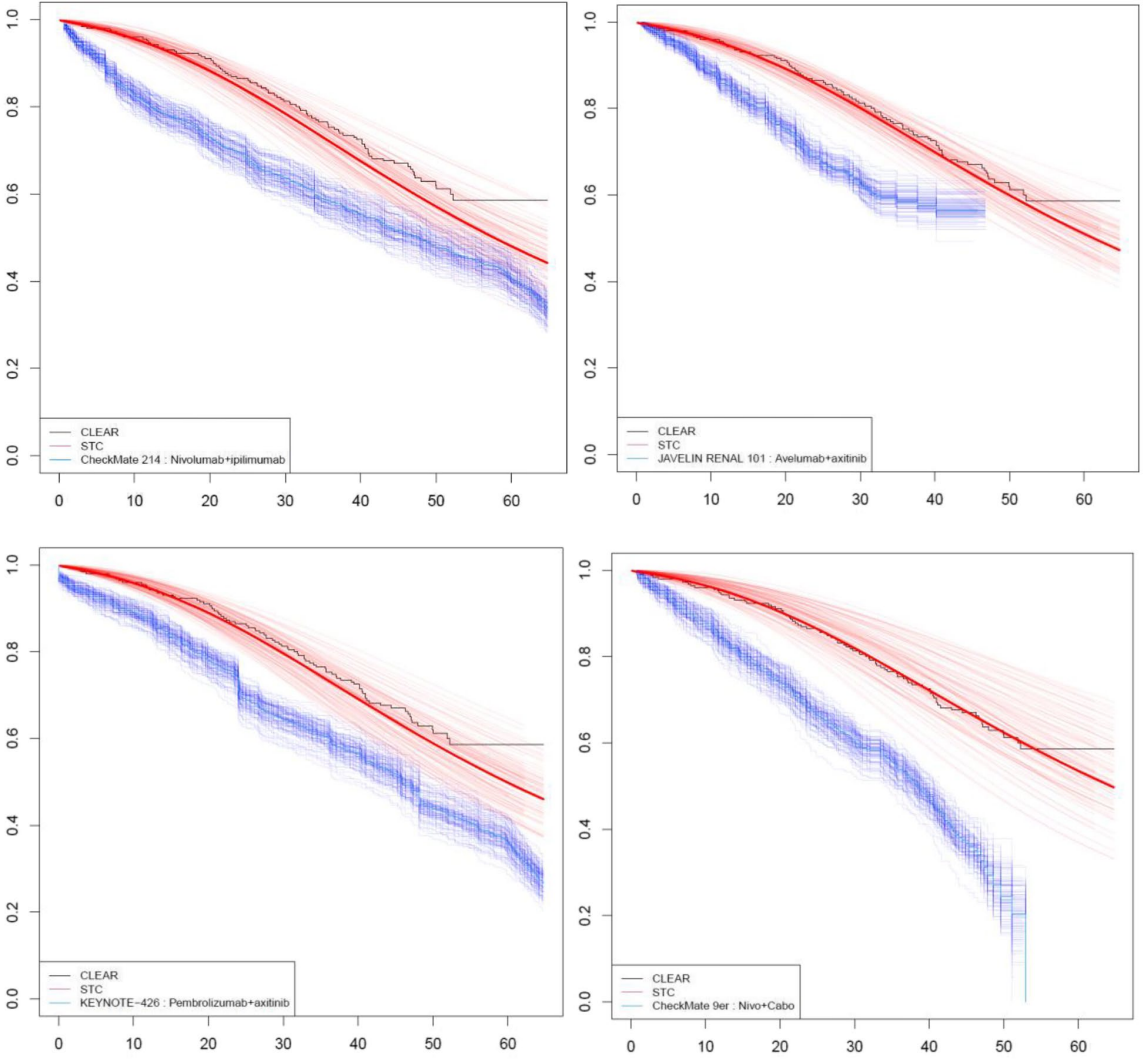
OS Comparison of covariate-adjusted 1 knot spline odds for LEN + PEM against comparators* * Compares LEN + PEM against KM curves for NIVO + IPI, AVE + AXI, PEM + AXI, and NIVO + CABO. Survival is measured in months, solid lines are base case STC estimates (of CLEAR survival adjusted to represent the comparator population) and KM estimates (from unadjusted trial data). Uncertainty is represented by 100 bootstrap resamples. AVE + AXI = avelumab + axitinib; KM = Kaplan–Meier; LEN + PEM = lenvatinib + pembrolizumab; NIVO + CABO = nivolumab + cabozantinib; OS = overall survival; PEM + AXI = pembrolizumab + axitinib; STC = simulated treatment comparison

Comparisons on mean difference in RMST for OS were favourable to LEN + PEM across all comparisons: 6.90 months (95% CI: 1.95, 11.36) versus NIVO + IPI; 5.31 months (95% CI: 3.58, 7.28) versus AVE + AXI; 5.99 months (95% CI: 1.82, 9.42) versus PEM + AXI; and 11.59 months (95% CI: 8.41, 15.38) versus NIVO + CABO (Table 3; Fig. 1). Hazard ratios estimated at 6, 12, and 18 months also indicated strong evidence of greater survival on LEN + PEM, although there was limited evidence of a difference in survival between LEN + PEM and NIVO + IPI and PEM + AXI at 24 months.

**Table 3:** OS (1 knot spline odds and naive) comparison of RMST* and hazard ratios (95% CI) at 6, 12, 18, and 24 months

Model	Trial	Comparator	Follow-up	RMST—LEN + PEM (95% CI)	RMST – comparator(95% CI)	Mean difference RMST (95% CI); (*p*-value)	6 months HR (95% CI)	12 months HR (95% CI)	18 months HR (95% CI)	24 months HR (95% CI)
1 knot spline odds	CheckMate 214^a^	NIVO + IPI	74.4 months	48.0 (43.4, 51.9)	41.1 (39.4, 43.1)	6.90 (1.95, 11.36) *p* = 0.02000	0.243 (0.144, 0.455) *p* = 0.00000	0.433 (0.297, 0.689) *p* = 0.00000	0.627 (0.431, 0.862) *p* = 0.00000	0.790 (0.576, 1.080) *p* = 0.16000
	JAVELIN RENAL^b^	AVE + AXI	46.7 months	39.5 (37.8, 41.1)	34.2 (32.7, 35.8)	5.31 (3.58, 7.28) *p* = 0.00000	0.343 (0.203, 0.534) *p* = 0.00000	0.401 (0.282, 0.563) *p* = 0.00000	0.481 (0.378, 0.690) *p* = 0.00000	0.620 (0.488, 0.818) *p* = 0.00000
	KEYNOTE-426^c^	PEM + AXI	73.7 months	47.4 (44.2, 50.6)	41.4 (39.1, 43.6)	5.99 (1.82, 9.42) *p* = 0.00000	0.359 (0.204, 0.669) *p* = 0.02000	0.510 (0.339, 0.813) *p* = 0.02000	0.611 (0.435, 0.949) *p* = 0.02000	0.695 (0.515, 1.012) *p* = 0.06000
	CheckMate 9ER^d^	NIVO + CABO	53.0 months	45.5 (42.8, 49.0)	33.9 (32.2, 35.9)	11.59 (8.41, 15.38) *p* = 0.00000	0.223 (0.108, 0.366) *p* = 0.00000	0.290 (0.153, 0.478) *p* = 0.00000	0.343 (0.192, 0.609) *p* = 0.00000	0.400 (0.243, 0.726) *p* = 0.00000
Naïve comparison (KM data only)	CheckMate 214^a^	NIVO + IPI	74.4 months	46.9 (44.4, 48.8)	41.1 (38.7, 43.5)	5.78 (2.26, 9.15) *p* = 0.00000	0.82 (0.40, 1.68) *p* = 0.58269	0.58 (0.38, 0.88) *p* = 0.0075389	0.65 (0.46, 0.92) *p* = 0.012807	0.63 (0.47, 0.84) *p* = 0.0011703
	JAVELIN RENAL^b^	AVE + AXI	46.7 months	37.5 (36.0, 38.6)	34.2 (33.1, 35.7)	3.317 (0.97, 4.66) *p* = 0.00000	1.47 (0.68, 3.20) *p* = 0.34478	0.71 (0.45, 1.12) *p* = 0.13595	1.10 (0.76, 1.59) *p* = 0.63012	0.86 (0.64, 1.16) *p* = 0.30634
	KEYNOTE-426^c^	PEM + AXI	73.7 months	46.9 (44.4, 49.0)	41.4 (38.9, 43.5)	5.478 (2.22, 8.23) *p* = 0.00000	0.52 (0.25, 1.09) *p *= 0.065802	0.72 (0.45, 1.15) *p* = 0.16043	1.07 (0.74, 1.57) *p* = 0.71524	1.05 (0.77, 1.43) *p* = 0.77656
	CheckMate 9ER^d^	NIVO + CABO	53.0 months	41.0 (39.4, 42.2)	33.9 (32.3, 35.5)	7.069 (4.63, 9.27) *p* = 0.00000	1.16 (0.53, 2.54) *p* = 0.71346	0.76 (0.48, 1.21) *p* = 0.24173	0.98 (0.66, 1.44) *p* = 0.90208	0.82 (0.60, 1.13) *p* = 0.22127

^*^RMST is measured in months up to 64.8 months CLEAR follow-up, or the follow-up of the comparator, whichever is shortest. *P*-values are one-sided

^a^Adjusted for AGE, REGION_ROW, PDL1_L1, ORGSGR1_GE2, LBONEN, LLYMPHN, LLIVEN, LLUNGN

^b^Adjusted for AGE, MSKCCP_FAVORABLE, IMDCP_FAVORABLE, ORGSGR1_GE2

^c^Adjusted for AGE, REGION_ROW, PDL1_L1, ORGSGR1_GE2, LBONEN, LLYMPHN, LLIVEN, LLUNGN

^d^Adjusted for AGE, REGION_ROW, MSKCCP_FAVORABLE, PDL1_L1, ORGSGR1_GE2, LBONEN, LLYMPHN, LLIVEN, LLUNGN

*AVE + AXI *avelumab + axitinib, *HR *hazard ratio, *KM *Kaplan–Meier, *LEN + PEM *lenvatinib + pembrolizumab, *NIVO + CABO* nivolumab + cabozantinib, *OS* overall survival, *PEM + AXI* pembrolizumab + axitinib, *RMST* Restricted Mean Survival Time

According to the naïve comparisons (i.e., KM data only), there was more limited evidence of a difference in survival at 6, 12, 18, and 24 months between LEN + PEM and all comparators, excluding NIVO + IPI at 12,18, and 24 months (Table 3). However, the results generally favoured LEN + PEM, at least numerically, on most comparisons. The mean difference in RMST also favoured LEN + PEM, however, the difference in survival was less pronounced than in the base case analysis.

### PFS

The maximum variance and AIC of the various survival models fit to PFS are summarised in Additional File 1, along with the 10 covariate-adjusted (log-logistic) models with lowest AIC. Similar to OS, there was limited difference in AIC between the models; the survival model with the lowest AIC was a log-logistic. The base case model selected for PFS was a log-logistic model, which adjusted for age, PDL1, MSKCC, IMDC, and number of metastatic sites ≥ 2 and all lesion locations (bone, lymph, liver).

**Fig. 2:**
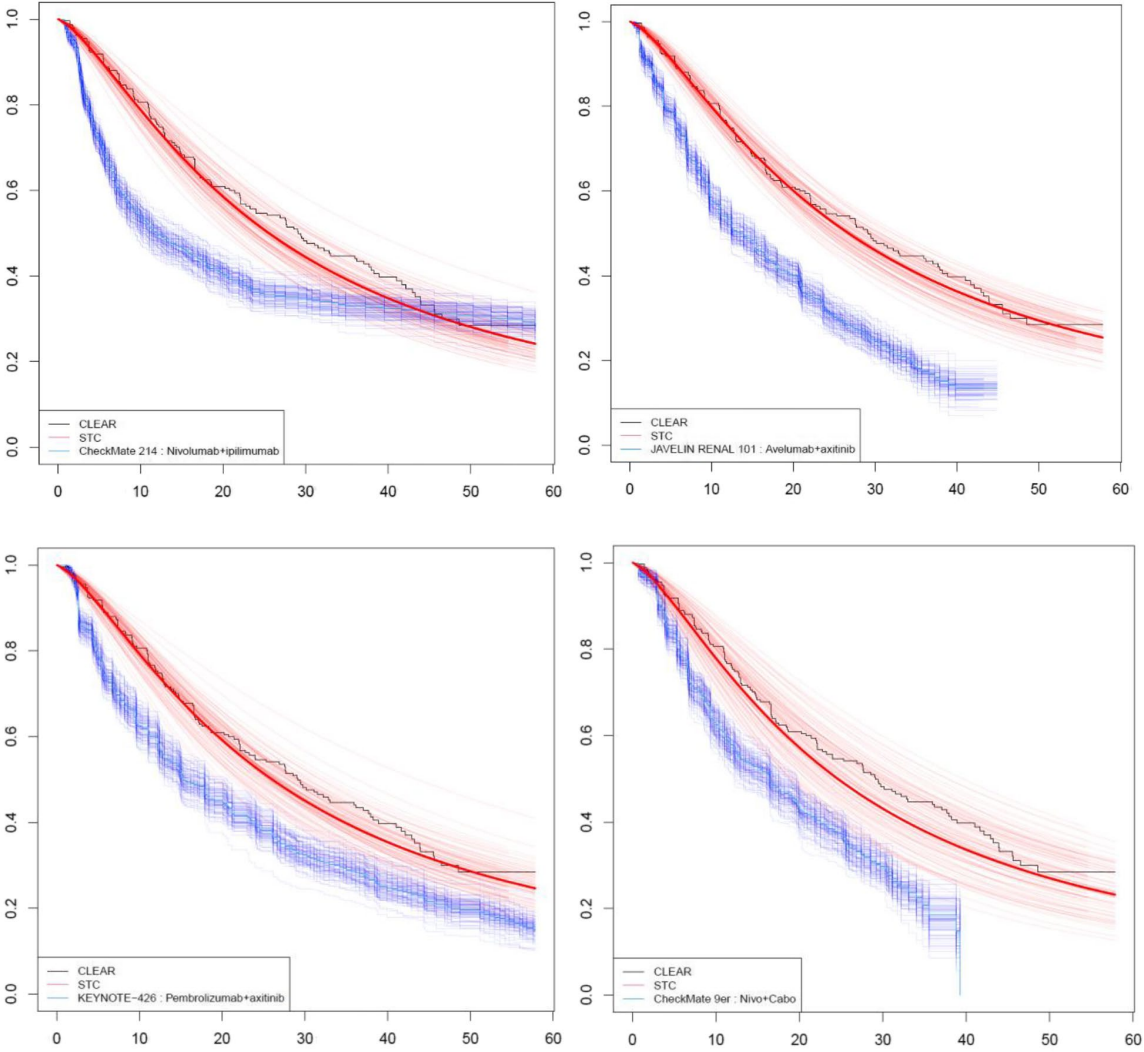
PFS Comparison of covariate-adjusted log-logistic for LEN + PEM against comparators* * Compares LEN + PEM against KM curves for NIVO + IPI, AVE + AXI, PEM + AXI, and NIVO + CABO. Survival is measured in months, solid lines are base case STC estimates (of CLEAR survival adjusted to represent the comparator population) and KM estimates (from unadjusted trial data). Uncertainty is represented by 100 bootstrap resamples. AVE + AXI = avelumab + axitinib; KM = Kaplan–Meier; LEN + PEM = lenvatinib + pembrolizumab; NIVO + CABO = nivolumab + cabozantinib; PEM + AXI = pembrolizumab + axitinib; PFS = progression-free survival; STC = simulated treatment comparison

Comparisons on RMST in PFS were also favourable to LEN + PEM across all comparisons: 4.50 months (95% CI: 0.92, 8.26) versus NIVO + IPI; 8.23 months (95% CI: 5.60, 10.57) versus AVE + AXI; 5.38 months (95% CI: 2.06, 9.09) versus PEM + AXI; and 4.58 months (95% CI: 0.09, 9.44) versus NIVO + CABO (Table 4; Fig. 2). Hazard ratios also indicated greater PFS on LEN + PEM on most comparisons, although lower survival was observed at 24 months versus NIVO + IPI. Comparisons at 12 and 18 months generally favoured LEN + PEM, with the exception of NIVO + IPI at 18 months, but there was limited evidence of a difference in PFS at this timepoint.

**Table 4:** PFS (log-logistic and naïve) comparison of RMST* and hazard ratios (95% CI) at 6, 12, 18, and 24 months

Model	Trial	Comparator	Follow-up	RMST—LEN + PEM(95% CI)	RMST– comparator(95% CI)	Mean difference RMST (95% CI); (*p*-value)	6 months HR (95% CI)	12 months HR (95% CI)	18 months HR (95% CI)	24 months HR (95% CI)
Log-logistic	CheckMate 214^a^	NIVO + IPI	70.3 months	29.2 (26.4, 32.0)	24.6 (22.7, 26.7)	4.50 (0.92, 8.26) *p* = 0.00000	0.434 (0.298, 0.598) *p* = 0.00000	0.756 (0.548, 1.144) *p* = 0.14000	1.034 (0.770, 1.468) *p* = 0.82000	1.596 (1.108, 2.308) *p* = 0.00000
	JAVELIN RENAL^b^	AVE + AXI	44.9 months	26.6 (24.2, 28.8)	18.4 (17.1, 19.9)	8.23 (5.60, 10.57) *p* = 0.00000	0.511 (0.381, 0.657) *p* = 0.00000	0.621 (0.496, 0.758) *p* = 0.00000	0.662 (0.503, 0.837) *p* = 0.00000	0.587 (0.445, 0.746) *p* = 0.00000
	KEYNOTE-246^c^	PEM + AXI	70.9 months	29.0 (25.7, 31.6)	23.7 (21.7, 25.6)	5.38 (2.06, 9.09) *p* = 0.00000	0.620 (0.461, 0.788) *p* = 0.00000	0.754 (0.587, 0.985) *p* = 0.04000	0.845 (0.669, 1.089) *p* = 0.24000	0.900 (0.695, 1.208) *p* = 0.54000
	CheckMate 9ER^d^	NIVO + CABO	39.3 months	23.8 (19.9, 28.4)	19.2 (17.9, 20.6)	4.58 (0.09, 9.44) *p* = 0.06000	0.607 (0.351, 1.036) *p* = 0.08000	0.756 (0.463, 1.134) *p* = 0.24000	0.838 (0.559, 1.262) *p* = 0.48000	0.681 (0.434, 1.103) *p* = 0.16000
Naïve comparison (KM data only)	CheckMate 214^a^	NIVO + IPI	70.3 months	28.5 (26.4, 31.7)	24.6 (22.6, 26.6)	3.88 (0.95, 6.80) *p* = 0.00000	0.93 (0.68, 1.28) *p* = 0.66684	0.61 (0.48, 0.78) *p* = 3.3944e-05	0.63 (0.51, 0.78) *p* = 8.8879e-06	0.64 (0.53, 0.78) *p* = 5.1791e-06
	JAVELIN RENAL^b^	AVE + AXI	44.9 months	25.4 (23.7, 27.2)	18.4 (17.0, 19.5)	7.06 (4.90, 9.219) *p* = 0.00000	0.93 (0.66, 1.31) *p* = 0.69000	0.66 (0.52, 0.85) *p* = 0.00090022	0.65 (0.52, 0.80) *p* = 5.0656e-05	0.65 (0.53, 0.80) *p* = 1.7483e-05
	KEYNOTE-246^c^	PEM + AXI	70.9 months	28.5 (26.1, 30.5)	23. 7 (21.6, 25.9)	4.866 (1.86, 7.78) *p* = 0.00000	0.91 (0.64, 1.27) *p* = 0.56319	0.63 (0.49, 0.81) *p* = 0.00030829	0.69 (0.55, 0.86) *p* = 0.00069869	0.74 (0.61, 0.91) *p* = 0.0038137
	CheckMate 9ER^d^	NIVO + CABO	39.3 months	23. 7 (22.3, 25.2)	19.3 (17.6, 20.5)	4.46 (2.79, 6.88) *p* = 0.00000	0.911 (0.63, 1.33) *p* = 0.62744	0.68 (0.52, 0.90) *p* = 0.0055019	0.67 (0.53, 0.85) *p* = 0.00071778	0.66 (0.53, 0.82) *p* = 0.00013588

^*^RMST is measured in months up to 57.82 months CLEAR follow-up, or the follow-up of the comparator, whichever is shortest. *P*-values are one-sided

^a^Adjusted for IMDCP_FAVORABLE, PDL1_L1, ORGSGR1_GE2, LBONEN, LLYMPHN, LLIVEN

^b^Adjusted for MSKCCP_FAVORABLE, IMDCP_FAVORABLE, ORGSGR1_GE2

^c^Adjusted for MSKCCP_FAVORABLE, IMDCP_FAVORABLE, PDL1_L1, ORGSGR1_GE2, LBONEN, LLYMPHN, LLIVEN

^d^Adjusted for IMDCP_FAVORABLE, PDL1_L1, ORGSGR1_GE2, LBONEN, LLYMPHN, LLIVEN

*AVE + AXI* avelumab + axitinib, *HR* hazard ratio, *KM* Kaplan–Meier, *LEN + PEM* lenvatinib + pembrolizumab, *NIVO + CABO* nivolumab + cabozantinib, *PEM + AXI* pembrolizumab + axitinib, *PFS* progression-free survival, *RMST* Restricted Mean Survival Time

In contrast, there was strong evidence of a difference in survival time at 12, 18, and 24 months in favour of LEN + PEM according to the naïve comparisons (i.e., KM data only) (Table 4). Estimated HRs at six months numerically favoured LEN + PEM, although there was limited evidence of a difference. The mean difference in RMST for PFS was aligned with the base case analysis. (Table 4).

### Sensitivity analysis

In sensitivity analysis we selected the next three models with lowest AIC, which included Weibull, 1-knot spline hazard, and gamma for OS, and 1-knot spline hazard, 1-knot spline normal, and exponential for PFS. Sensitivities for both OS and PFS were also conducted using the base case survival model with all covariates, no covariates, and a naive comparison of KM curve, fully aligning with NICE and published guidelines [6, 7]. With the exception of the all covariates model, there was limited model uncertainty, whether from choice of covariates or choice of survival distribution (see Additional file 2). In the all covariates models, three of the four comparisons on mean difference in OS were no longer significant, along with two of the four comparisons on mean difference in PFS.

## Discussion

We developed and applied a novel methodology for comparing survival outcomes when treatments are disconnected or comparator arms are considered dissimilar (i.e., in unanchored settings), which can be applied to any disease area or setting. In our application to LEN + PEM for advanced RCC, we found that LEN + PEM was associated with greater OS and PFS across most comparisons with other first-line treatment regimens. We conducted a range of sensitivity analyses including using alternative survival models with the next lowest AIC, as well as including all covariates and no covariates in separate regression analyses. Consistent with NICE and published guidelines, we also used only KM data in a naïve comparison [6, 7]. The difference in results between the base case and naïve comparison was more pronounced on OS, where results more strongly favoured LEN + PEM in the base case analysis. In contrast, there was more limited evidence of a difference in survival on PFS in the base case analysis than in the naïve comparison, highlighting the importance of adjusting for covariates in STC. Furthermore, these differences in OS and PFS between the STC and naïve comparison may bolster confidence in the methodology to produce less biased estimates than other approaches, as observed in anchored settings [20]. Across most sensitivity analyses, the results remained broadly unchanged. In the all covariates models, results were often sensitive to covariate selections. However, this may be simply a consequence of including too many covariates in the STC. As with all STC in unanchored settings, the analysis may be biased by unmeasured confounders, due to not including all potential prognostic factors and effect modifiers, for example [6]. However, there was limited model uncertainty across all considered analyses, reducing the potential impact of unreported baseline characteristics.

In a previously published NMA that compared LEN + PEM with the same first-line treatment comparators considered here in advanced RCC, the authors found some evidence in favour of LEN + PEM on OS and PFS [37]. For instance, the authors found LEN + PEM showed a > 80% probability of providing greater PFS benefit over all available comparators, with a significant benefit observed in 14 out of 18 comparators, including NIVO + IP (HR = 0.44, 95%CrI 0.23–0.82). These results were broadly consistent with our analysis at six months (HR = 0.43, 95% CI 0.30, 0.60). The findings from our analysis that LEN + PEM was strongly associated with greater OS and PFS across most comparisons highlight the importance of assessing the extent that comparator arms in trials may be dissimilar. At the time of the analysis, a substantially higher proportion of patients in the control arm of CLEAR than comparator trials received subsequent anti-PD-1/PD-L1 inhibitors. This biased comparisons anchored on SUN against LEN + PEM due to the greater survival advantage observed in the control group in CLEAR. As such, the benefit observed on OS and PFS in the NMA may have been understated in a number of comparisons relative to our analysis.

To date, population-adjusted methods for indirect comparison of survival outcomes have almost exclusively relied on the use of MAIC [19]. However, MAIC using Cox analysis relies on the assumption of proportional hazards; that is, it assumes the relative hazard of survival remains constant over time. This is likely an over-simplification for survival outcomes, for which treatment effects typically vary over time. The approach is also limited in its use to inform HTAs of treatments designed to prolong patients’ life expectancy as it does not extrapolate beyond observed trial data [44]. This further limits its use in economic models designed to evaluate lifetime costs and consequences of different treatments. A major advantage of STC is that it overcomes the assumption of proportional hazards by fitting parametric models, including Royston Parmar spline models, which can be used to provide extrapolations for use in economic models. Our findings suggest STC may be used to guide clinical decision-making in unanchored settings and, importantly, inform HTAs and economic models. A comprehensive simulation study is recommended as further research to support and corroborate these findings.

### Limitations

There are a number of limitations associated with the use of population-adjusted methods in unanchored settings that extend to our STC. The primary limitation is the loss of randomisation when comparing treatments that are dissimilar/disconnected; that is, the control group is no longer used so the randomisation property is lost. The validity of comparisons is therefore reliant on regression models including all important effect modifiers and prognostic variables, and having sufficient data to make reliable predictions of survival or hazard in comparator populations [6]. However, in unanchored settings, it is almost impossible to know if all prognostic factors and effect modifiers have been included and if the model is correctly specified, which is often a criticism of the approach by regulatory bodies [6]. The regression models also do not account for variability in the target population and only predict at average covariate values. Technically, due to the non-linearity of survival function, the survival probability at the mean covariate values does not equal the mean survival probability in the target population. However, the former usually provides a reasonable approximation of the latter in practice, although statistical simulation may be warranted to quantify the bias. We considered all possible covariates, which were those recorded by CLEAR and reported by at least one comparator study. We also explored multiple regression models and found limited potential impact of this form of model misspecification. Again, a comprehensive simulations study is warranted to support this finding and assess the effect of a prognostic factor that may not be reported in any of the included trials. Such a study, or application to historical examples where long-term data are available against which to test extrapolations from older short-term data, is recommended as further research to support and corroborate these findings.

Another limitation of population-adjusted methods is that the target population for comparisons is that of the comparator trial/treatment rather than trial/treatment of interest. In the case of our analysis, the comparisons were based on the population of comparator treatments rather than the population for LEN + PEM in CLEAR. As such, there may have been some important loss of information from CLEAR as only baseline characteristics that could be matched to the comparator population were used in the analysis. The recent method of multilevel network meta-regression (ML-NMR) extends STC to allow comparison in any target population, but has only been developed for anchored comparisons and has not yet been applied to survival outcomes [8, 51]. A further limitation is that comparisons with other treatments were limited to pairwise comparisons since the reporting of baseline characteristics (i.e., prognostic factors and effect modifiers) varied across trials and had to be matched precisely to the IPD; this is a general criticism of population-adjusted methods. A final theoretical limitation is that our hazard ratios do not imply a causal relationship between differences in treatment and differences in outcome. Using STC to estimate a causal hazard difference could be worthwhile for future research [52].

Although simulation studies have shown that MAIC is sensitive to model misspecification and STC produces less biased estimates than MAIC in the anchored setting, it remains unclear how well STC performs next to MAIC in unanchored settings. A comprehensive simulation study is needed to compare the predictive performance of MAIC and STC, as well as ML-NMR, in unanchored settings.

While STC provides extrapolation options for the treatment of interest, extrapolations for comparator treatments must be generated by fitting independent survival models to their KM data. We did not extrapolate beyond the CLEAR trial but would need long-term external data to validate such extrapolations, and this validation would need to be reflected in choice of survival models.

By removing the common comparator and analysing relative effects in a unanchored setting, we’ve eliminated the imbalance in the use of rescue therapies in the control arm of the included studies. There may remain imbalance in the use of rescue therapies in the treatment arms that were not accounted for in the present study. However, it is unlikely that any rescue therapy involving immunotherapy would have conferred a survival advantage in the treatment arms due to prior use of immunotherapy. Nevertheless, this remains a limitation of the present study.

## Conclusions

We developed and applied novel methodology for comparing survival outcomes when treatments are disconnected or dissimilar (i.e., in unanchored settings) using STC, which can be adapted to any disease area or setting. The methodology offers a number of key advantages over other unanchored population-adjusted indirect treatment comparison methods (e.g., MAIC) by overcoming the assumption of proportional hazards and fitting alternative parametric survival models, which can be used to guide clinical decision-making in relation to patients’ longer-term survival outcomes. More importantly to HTAs and economic models (and, hence, regulatory bodies), STC can be used to provide extrapolation options to examine lifetime costs and consequences associated with new or existing treatments.

In our application to LEN + PEM in advanced RCC, we found that LEN + PEM was associated with greater OS and PFS compared with other first-line immunotherapy combination treatments, including dual-type combination and kinase inhibitor combination treatments. Our analysis highlights the importance of using unanchored comparisons when control arms differ across trials. In the case of LEN + PEM, the control group in CLEAR was different from the control group in comparator trials due to a high proportion of patients (whom discontinued treatment) receiving subsequent PD-1 or PD-L1 inhibitor therapy, giving a survival advantage to patients in this group relative to comparator trials. Our findings suggest any comparison anchored on the control group would bias against LEN + PEM.

## Supplementary Information

Supplementary Material 1.

Supplementary Material 2.

## Data Availability

The primary trial data analysed during the current study are not publicly available due to containing sensitive patient information but the generated data using published evidence and associated code used to run all analysed are available from the corresponding author on reasonable request.

## Abbreviations

AIC: Akaike information criterion
AXI: Axitinib
AVE: Avelumab
CABO: Cabozantinib
HTA: Health technology assessment
IMDC: International Metastatic RCC Database Consortium
IPD: Individual patient data
IPI: Ipilimumab
ITC: Indirect treatment comparison
KM: Kaplan-Meier
LEN: Lenvatinib
MAIC: Matching-adjusted indirect comparison
ML-NMR: Multi-level network meta-regression
MSKCC: Memorial Sloan Kettering Cancer Center
NICE: National institute for Health and Care Excellence
NIVO: Nivolumab
NMA: Network meta-analysis
OS: Overall survival
PEM: Pembrolizumab
PFS: Progression-free survival
RCC: Renal cell carcinoma
RMST: Restricted mean survival time
RTK: Receptor tyrosine kinases
SUN: Sunitinib
STC: Simulated Treatment Comparison
VEGF: Vascular endothelial growth factor
