# Supplementary material for: Unanchored simulated treatment comparison on survival outcomes using parametric and Royston-Parmar models with application to lenvatinib plus pembrolizumab in renal cell carcinoma
Source: BMC Med Res Methodol. 2025 Jan 30;25:26. doi: 10.1186/s12874-025-02480-x (PMC11780865; doi:10.1186/s12874-025-02480-x)
Supplement: Supplementary file 1 — Supplementary Material 1. [file 12874_2025_2480_MOESM1_ESM.docx]

# Unanchored Simulated Treatment Comparison on survival outcomes using parametric and Royston-Parmar models with application to lenvatinib plus pembrolizumab in renal cell carcinoma

## Additional file 1

## Table of contents

[Table S1 OS Comparison of survival models for STC 2](#_Toc152319459)

[Table S2 OS Comparison of covariate adjusted 1 knot spline odds models for STC* 2](#_Toc152319460)

[Table S3 PFS Comparison of survival models for STC 4](#_Toc152319461)

[Table S4 PFS Comparison of covariate adjusted log-logistic models for STC* 4](#_Toc152319462)

Overall, there was little difference in AIC between the models, although the survival model with the lowest AIC was a 1-knot spline odds. The maximum variance was large for the 3-knot spline models, indicating convergence issues, while the 1-knot spline normal model did not converge.

Table S1 OS Comparison of survival models for STC

| **Model** | **Max variance** | **AIC** |
| --- | --- | --- |
| Exponential | 0.01 | 1612.29 |
| Weibull | 0.01 | 1600.24 |
| Gompertz | 0.03 | 1601.79 |
| Gamma | 0.03 | 1601.18 |
| Log-logistic | 0.01 | 1602.22 |
| Log-normal | 0.01 | 1619.89 |
| Generalised-F | 58.24 | 1601.69 |
| 1-knot spline hazard | 0.32 | 1600.90 |
| 2-knot spline hazard | 0.28 | 1601.80 |
| 3-knot spline hazard | 3.12 | 1602.32 |
| 1-knot spline odds | 0.29 | 1599.65 |
| 2-knot spline odds | 0.32 | 1601.62 |
| 3-knot spline odds | 4.40 | 1602.32 |
| 1-knot spline normal | NA | NA |
| 2-knot spline normal | 0.10 | 1602.16 |
| 3-knot spline normal | 1.52 | 1602.40 |

AIC=Akaike information criterion; STC=simulated treatment comparison

The base case model selected for PFS was a log-logistic model, which adjusted for age, PDL1, MSKCC, IMDC, and number of metastatic sites ≥2 and all lesion locations (bone, lymph, liver). As with OS, the models varied by comparator as we only adjusted for variables reported by comparator trials.

Table S2 OS Comparison of covariate adjusted 1 knot spline odds models for STC*

| **Survival formula** | **N covariates** | **Max variance** | **AIC** |
| --- | --- | --- | --- |
| AGE + IMDCP_FAVORABLE + LBONEN + LLYMPHN + LLIVEN + LLUNGN | 6 | 0.88 | 1568.80 |
| AGE + IMDCP_FAVORABLE + LLYMPHN + LLIVEN + LLUNGN | 5 | 0.87 | 1569.33 |
| AGE + MSKCCP_FAVORABLE + IMDCP_FAVORABLE + LBONEN + LLYMPHN + LLIVEN + LLUNGN | 7 | 0.88 | 1569.34 |
| AGE + IMDCP_FAVORABLE + LBONEN + LLYMPHN + LLIVEN | 5 | 0.80 | 1569.69 |
| AGE + MSKCCP_FAVORABLE + IMDCP_FAVORABLE + LLYMPHN + LLIVEN + LLUNGN | 6 | 0.88 | 1569.96 |
| AGE + MSKCCP_FAVORABLE + LBONEN + LLYMPHN + LLIVEN + LLUNGN | 6 | 0.88 | 1570.02 |
| AGE + MSKCCP_FAVORABLE + IMDCP_FAVORABLE + LBONEN + LLYMPHN + LLIVEN | 6 | 0.81 | 1570.21 |
| AGE + IMDCP_FAVORABLE + LLYMPHN + LLIVEN | 4 | 0.80 | 1570.35 |
| AGE + SEXN + IMDCP_FAVORABLE + LBONEN + LLYMPHN + LLIVEN + LLUNGN | 7 | 0.98 | 1570.79 |
| AGE + IMDCP_FAVORABLE + PDL1_L1 + LBONEN + LLYMPHN + LLIVEN + LLUNGN | 7 | 0.88 | 1570.80 |

* PDL1_L1 = PDL1<1%, ORGSGR1_GE2= number of metastatic sites ≥2

AIC=Akaike information criterion; STC=simulated treatment comparison

Similar to OS, there was limited difference in AIC between the models; the survival model with the lowest AIC was a log-logistic. The maximum variance was very high only for the Generalised-F model.

Table S3 PFS Comparison of survival models for STC

| **Model** | **Max variance** | **AIC** |
| --- | --- | --- |
| Exponential | 0.00 | 1882.72 |
| Weibull | 0.00 | 1883.53 |
| Gompertz | 0.01 | 1884.72 |
| Gamma | 0.02 | 1882.84 |
| Log-logistic | 0.01 | 1880.84 |
| Log-normal | 0.01 | 1884.00 |
| Generalised-F | 161.63 | 1883.48 |
| 1-knot spline hazard | 0.11 | 1881.35 |
| 2-knot spline hazard | 0.12 | 1883.39 |
| 3-knot spline hazard | 0.17 | 1885.24 |
| 1-knot spline odds | 0.11 | 1882.83 |
| 2-knot spline odds | 0.13 | 1884.31 |
| 3-knot spline odds | 0.26 | 1885.88 |
| 1-knot spline normal | 0.02 | 1881.78 |
| 2-knot spline normal | 0.02 | 1883.68 |
| 3-knot spline normal | 0.08 | 1885.47 |

AIC=Akaike information criterion; STC=simulated treatment comparison

Table S4 PFS Comparison of covariate adjusted log-logistic models for STC*

| **Survival formula** | **N covariates** | **Max variance** | **AIC** |
| --- | --- | --- | --- |
| AGE + ORGSGR1_GE2 | 2 | 0.29 | 1287.21 |
| AGE + IMDCP_FAVORABLE + ORGSGR1_GE2 | 3 | 0.30 | 1288.04 |
| AGE + ORGSGR1_GE2 + LLUNGN | 3 | 0.31 | 1288.08 |
| AGE + MSKCCP_FAVORABLE + ORGSGR1_GE2 | 3 | 0.30 | 1288.75 |
| AGE + ORGSGR1_GE2 + LLIVEN | 3 | 0.30 | 1289.01 |
| AGE + ORGSGR1_GE2 + LBONEN | 3 | 0.29 | 1289.01 |
| ORGSGR1_GE2 | 1 | 0.03 | 1289.02 |
| AGE + ORGSGR1_GE2 + LLYMPHN | 3 | 0.32 | 1289.02 |
| AGE + IMDCP_FAVORABLE + ORGSGR1_GE2 + LLUNGN | 4 | 0.32 | 1289.06 |
| AGE + PDL1_L1 + ORGSGR1_GE2 | 3 | 0.29 | 1289.07 |

* PDL1_L1 = PDL1<1%, ORGSGR1_GE2= number of metastatic sites ≥2

AIC=Akaike information criterion; STC=simulated treatment comparison
