# Supplementary material for: Unanchored simulated treatment comparison on survival outcomes using parametric and Royston-Parmar models with application to lenvatinib plus pembrolizumab in renal cell carcinoma
Source: BMC Med Res Methodol. 2025 Jan 30;25:26. doi: 10.1186/s12874-025-02480-x (PMC11780865; doi:10.1186/s12874-025-02480-x)
Supplement: Supplementary file 2 — Supplementary Material 2. [file 12874_2025_2480_MOESM2_ESM.docx]

# Unanchored Simulated Treatment Comparison on survival outcomes using parametric and Royston-Parmar models with application to lenvatinib plus pembrolizumab in renal cell carcinoma

## Additional file 2

## Table of contents

[Table S1 OS comparison of mean survival* and hazard ratios (95% CI) at 6, 12, 18, and 24 months across – base case and sensitivity analyses 2](#_Toc152319494)

[Table S2 PFS comparison of mean survival* and hazard ratios (95% CI) at 6, 12, 18, and 24 months – base case and sensitivity analyses 5](#_Toc152319495)

Table S1 OS comparison of mean survival* and hazard ratios (95% CI) at 6, 12, 18, and 24 months across – base case and sensitivity analyses

| **Model** | **Trial** | **Comparator** | **Follow-up** | **Mean survival - LEN+PEM**  (95% CI) | **Mean survival – comparator (95% CI)** | **Mean difference (95% CI); (p-value)** | **6 months HR (95% CI)** | **12 months HR (95% CI)** | **18 months HR (95% CI)** | **24 months HR (95% CI)** |
| --- | --- | --- | --- | --- | --- | --- | --- | --- | --- | --- |
| **Base case analysis** | CheckMate 214^a^ | NIVO+IPI | 74.4 months | 46.99 (43.36, 50.11) | 41.10 (38.70, 43.45) | 5.90 (0.99, 10.08) p=0.020202 | 0.26 (0.16, 0.47) p=0.00000 | 0.46 (0.32, 0.70) p=0.00000 | 0.66 (0.48, 0.96) p=0.02000 | 0.87 (0.63, 1.17) p=0.28000 |
|  | JAVELIN^b^ | AVE+AXI | 46.7 months | 39.38 (37.61, 40.82) | 34.22 (33.12, 35.72) | 5.17 (2.56, 7.19) p=0.00000 | 0.33 (0.23, 0.50) p=0.00000 | 0.40 (0.28, 0.58) p=0.00000 | 0.48 (0.36, 0.68) p=0.00000 | 0.60 (0.47, 0.85) p=0.00000 |
|  | KEYNOTE-426^c^ | PEM+AXI | 73.7 months | 47.70 (43.88, 51.04) | 41.41 (38.93, 43.54) | 6.29 (2.20, 10.70) p=0.00000 | 0.34 (0.19, 0.64) p=0.02000 | 0.49 (0.31, 0.70) p=0.00000 | 0.60 (0.43, 0.83) p=0.00000 | 0.70 (0.52, 0.93) p=0.02000 |
|  | CheckMate 9ER^d^ | NIVO+CABO | 53.0 months | 43.87 (40.66, 47.78) | 33.91 (32.27, 35.52) | 9.97 (6.37, 14.13) p=0.00000 | 0.26 (0.12, 0.49) p=0.00000 | 0.35 (0.18, 0.61) p=0.00000 | 0.43 (0.23, 0.70) p=0.00000 | 0.49 (0.28, 0.76) p=0.02000 |
| **1 knot spline odds all covariates** | CheckMate 214^a^ | NIVO+IPI | 74.4 months | 44.13 (34.62, 52.089) | 41.10 (38.70, 43.45) | 3.03 (-5.24, 10.47) p=0.50000 | 0.31 (0.15, 0.58) p=0.02000 | 0.54 (0.29, 0.97) p=0.02000 | 0.76 (0.45, 1.40) p=0.48000 | 0.95 (0.55, 1.72) p=0.92000 |
|  | JAVELIN^b^ | AVE+AXI | 46.7 months | 37.71 (33.25, 41.53) | 34.22 (33.12, 35.72) | 3.50 (-1.767, 7.225) p=0.14141 | 0.45 (0.21, 0.87) p=0.02000 | 0.55 (0.29, 0.96) p=0.02000 | 0.67 (0.34, 1.09) p=0.16000 | 0.80 (0.44, 1.33) p=0.48000 |
|  | KEYNOTE-426^c^ | PEM+AXI | 73.7 months | 45.14 (37.40, 50.89) | 41.41 (38.93, 43.54) | 3.73 (-4.55, 9.96) p=0.44000 | 0.48 (0.22, 1.11) p=0.12000 | 0.65 (0.37, 1.23) p=0.24000 | 0.77 (0.45, 1.38) p=0.42000 | 0.88 (0.54, 1.58) p=0.68000 |
|  | CheckMate 9ER^d^ | NIVO+CABO | 53.0 months | 41.91 (34.92, 47.93) | 33.91 (32.27, 35.52) | 8.00 (0.76, 14.12) p=0.04000 | 0.36 (0.13, 0.92) p=0.04000 | 0.49 (0.19, 1.15) p=0.10000 | 0.57 (0.24, 1.25) p=0.16000 | 0.64 (0.28, 1.34) p=0.22000 |
| **1 knot spline odds no covariates** | CheckMate 214^a^ | NIVO+IPI | 74.4 months | 45.32 (43.18, 46.83) | 41.10 (38.70, 43.45) | 4.21 (0.97, 6.83) p=0.020202 | 0.38 (0.25, 0.55) p=0.00000 | 0.61 (0.45, 0.82) p=0.00000 | 0.80 (0.63, 1.05) p=0.16000 | 0.93 (0.75, 1.23) p=0.66000 |
|  | JAVELIN^b^ | AVE+AXI | 46.7 months | 37.45 (36.06, 38.60) | 34.22 (33.12, 35.72) | 3.24 (1.13, 4.60) p=0.020202 | 0.54 (0.37, 0.77) p=0.00000 | 0.59 (0.46, 0.81) p=0.00000 | 0.66 (0.52, 0.88) p=0.00000 | 0.78 (0.59, 1.04) p=0.10000 |
|  | KEYNOTE-426^c^ | PEM+AXI | 73.7 months | 45.32 (42.89, 46.83) | 41.41 (38.93, 43.54) | 3.91 (0.40, 6.85) p=0.04000 | 0.56 (0.39, 0.94) p=0.06000 | 0.70 (0.51, 1.02) p=0.08000 | 0.78 (0.61, 0.98) p=0.02000 | 0.84 (0.69, 1.04) p=0.22000 |
|  | CheckMate 9ER^d^ | NIVO+CABO | 53.0 months | 40.82 (38.98, 42.43) | 33.91 (32.27, 35.52) | 6.91 (4.55, 9.22) p=0.00000 | 0.47 (0.32, 0.70) p=0.00000 | 0.58 (0.43, 0.74) p=0.00000 | 0.64 (0.52, 0.81) p=0.00000 | 0.70 (0.53, 0.89) p=0.00000 |
| **Weibull** | CheckMate 214^a^ | NIVO+IPI | 74.4 months | 47.27 (43.99, 50.63) | 41.10 (38.70, 43.45) | 6.16 (2.17, 10.03) p=0.00000 | 0.32 (0.22, 0.46) p=0.00000 | 0.52 (0.38, 0.69) p=0.00000 | 0.67 (0.50, 0.90) p=0.02000 | 0.79 (0.60, 1.07) p=0.14000 |
|  | JAVELIN^b^ | AVE+AXI | 46.7 months | 39.19 (37.82, 40.62) | 34.22 (33.12, 35.72) | 4.97 (2.92, 7.07) p=0.00000 | 0.44 (0.23, 0.67) p=0.00000 | 0.50 (0.32, 0.68) p=0.00000 | 0.54 (0.42, 0.69) p=0.00000 | 0.64 (0.45, 0.85) p=0.00000 |
|  | KEYNOTE-426^c^ | PEM+AXI | 73.7 months | 48.00 (43.76, 50.99) | 41.41 (38.93, 43.54) | 6.59 (2.12, 9.77) p=0.00000 | 0.41 (0.244, 0.93) p=0.00000 | 0.59 (0.36, 0.83) p=0.00000 | 0.63 (0.45, 0.86) p=0.00000 | 0.68 (0.49, 0.90) p=0.04000 |
|  | CheckMate 9ER^d^ | NIVO+CABO | 53.0 months | 43.92 (38.96, 48.80) | 33.91 (32.27, 35.52) | 10.01 (4.74, 14.55) p=0.00000 | 0.33 (0.18, 0.68) p=0.00000 | 0.42 (0.24, 0.73) p=0.00000 | 0.47 (0.27, 0.86) p=0.00000 | 0.49 (0.27, 0.91) p=0.04000 |
| **1-knot spline hazard** | CheckMate 214^a^ | NIVO+IPI | 74.4 months | 47.37 (43.43, 51.71) | 41.10 (38.70, 43.45) | 6.26 (1.20, 11.32) p=0.00000 | 0.26 (0.17, 0.45) p=0.00000 | 0.44 (0.31, 0.63) p=0.00000 | 0.60 (0.45, 0.79) p=0.02000 | 0.75 (0.59, 0.99) p=0.04000 |
|  | JAVELIN^b^ | AVE+AXI | 46.7 months | 39.48 (37.68, 40.93) | 34.22 (33.12, 35.72) | 5.26 (2.85, 7.02) p=0.00000 | 0.36 (0.24, 0.58) p=0.00000 | 0.40 (0.29, 0.63) p=0.00000 | 0.47 (0.37, 0.68) p=0.00000 | 0.59 (0.45, 0.80) p=0.00000 |
|  | KEYNOTE-426^c^ | PEM+AXI | 73.7 months | 48.10 (44.61, 50.73) | 41.41 (38.93, 43.54) | 6.69 (1.70, 10.40) p=0.00000 | 0.37 (0.22, 0.76) p=0.00000 | 0.49 (0.34, 0.81) p=0.02000 | 0.56 (0.38, 0.74) p=0.00000 | 0.64 (0.44, 0.84) p=0.00000 |
|  | CheckMate 9ER^d^ | NIVO+CABO | 53.0 months | 44.12 (40.07, 48.31) | 33.91 (32.27, 35.52) | 10.21 (5.21, 14.41) p=0.00000 | 0.28 (0.16, 0.56) p=0.00000 | 0.33 (0.20, 0.62) p=0.00000 | 0.40 (0.24, 0.68) p=0.00000 | 0.46 (0.29, 0.77) p=0.02000 |
| **Gamma** | CheckMate 214^a^ | NIVO+IPI | 74.4 months | 47.17 (44.03, 51.13) | 41.10 (38.70, 43.45) | 6.07 (2.10, 10.77) p=0.00000 | 0.31 (0.18, 0.52) p=0.00000 | 0.54 (0.39, 0.74) p=0.00000 | 0.70 (0.54, 0.93) p=0.04000 | 0.81 (0.65, 1.10) p=0.20000 |
|  | JAVELIN^b^ | AVE+AXI | 46.7 months | 39.05 (37.34, 40.55) | 34.22 (33.12, 35.72) | 4.83 (2.27, 6.62) p=0.00000 | 0.43 (0.26, 0.74) p=0.00000 | 0.51 (0.35, 0.67) p=0.00000 | 0.56 (0.39, 0.74) p=0.00000 | 0.64 (0.44, 0.86) p=0.00000 |
|  | KEYNOTE-426^c^ | PEM+AXI | 73.7 months | 47.88 (44.74, 52.04) | 41.41 (38.93, 43.54) | 6.47 (2.10, 10.12) p=0.00000 | 0.45 (0.28, 0.86) p=0.00000 | 0.60 (0.43, 0.86) p=0.02000 | 0.68 (0.50, 0.90) p=0.02000 | 0.72 (0.54, 0.94) p=0.02000 |
|  | CheckMate 9ER^d^ | NIVO+CABO | 53.0 months | 43.89 (39.88, 47.81) | 33.91 (32.27, 35.52) | 9.99 (6.10, 14.53) p=0.00000 | 0.34 (0.17, 0.67) p=0.00000 | 0.43 (0.28, 0.72) p=0.00000 | 0.48 (0.28, 0.77) p=0.00000 | 0.51 (0.28, 0.87) p=0.00000 |
| **KM only** | CheckMate 214^a^ | NIVO+IPI | 74.4 months | 46.89 (44.41, 48.79) | 41.10 (38.70, 43.45) | 5.78 (2.26, 9.15) p=1.00000 | 0.82 (0.40, 1.68) p=0.58269 | 0.58 (0.38, 0.88) p=0.0075389 | 0.65 (0.46, 0.92) p=0.012807 | 0.63 (0.47, 0.84) p=0.0011703 |
|  | JAVELIN^b^ | AVE+AXI | 46.7 months | 37.53 (35.99, 38.56) | 34.22 (33.12, 35.72) | 3.32 (0.97, 4.66) p=1.00000 | 1.47 (0.68, 3.20) p=0.34478 | 0.71 (0.45, 1.12) p=0.13595 | 1.10 (0.76, 1.59) p=0.63012 | 0.86 (0.64, 1.16) p=0.30634 |
|  | KEYNOTE-426^c^ | PEM+AXI | 73.7 months | 46.89 (44.44, 49.00) | 41.41 (38.93, 43.54) | 5.48 (2.22, 8.23) p=1.00000 | 0.52 (0.25, 1.09) p=0.065802 | 0.72 (0.45, 1.15) p=0.16043 | 1.07 (0.74, 1.57) p=0.71524 | 1.05 (0.77, 1.43) p=0.77656 |
|  | CheckMate 9ER^d^ | NIVO+CABO | 53.0 months | 40.98 (39.40, 42.22) | 33.91 (32.27, 35.52) | 7.07 (4.63, 9.27) p=1.00000 | 1.16 (0.53, 2.54) p=0.71346 | 0.76 (0.48, 1.21) p=0.24173 | 0.98 (0.66, 1.44) p=0.90208 | 0.82 (0.60, 1.13) p=0.22127 |

* RMST is measured in months up to 64.8 months CLEAR follow-up, or the follow-up of the comparator, whichever is shortest. P-values are one-sided.

a Adjusted for AGE, REGION_ROW, PDL1_L1, ORGSGR1_GE2, LBONEN, LLYMPHN, LLIVEN, LLUNGN

b Adjusted for AGE, MSKCCP_FAVORABLE, IMDCP_FAVORABLE, ORGSGR1_GE2

c Adjusted for AGE, REGION_ROW, PDL1_L1, ORGSGR1_GE2, LBONEN, LLYMPHN, LLIVEN, LLUNGN

d Adjusted for AGE, REGION_ROW, MSKCCP_FAVORABLE, PDL1_L1, ORGSGR1_GE2, LBONEN, LLYMPHN, LLIVEN, LLUNGN

AVE+AXI=avelumab + axitinib; HR=hazard ratio; KM=Kaplan-Meier; LEN+PEM=lenvatinib + pembrolizumab; NIVO+CABO=nivolumab + cabozantinib; OS=overall survival; PEM+AXI=pembrolizumab + axitinib; RMST=Restricted Mean Survival Time

Table S2 PFS comparison of mean survival* and hazard ratios (95% CI) at 6, 12, 18, and 24 months – base case and sensitivity analyses

| **Model** | **Trial** | **Comparator** | **Follow-up** | **Mean survival - LEN+PEM**  (95% CI) | **Mean survival – comparator (95% CI)** | **Mean difference (95% CI); (p-value)** | **6 months HR (95% CI)** | **12 months HR (95% CI)** | **18 months HR (95% CI)** | **24 months HR (95% CI)** |
| --- | --- | --- | --- | --- | --- | --- | --- | --- | --- | --- |
| **Base case analysis** | CheckMate 214^a^ | NIVO+IPI | 70.3 months | 29.03 (25.65, 32.07) | 24.64 (22.62, 26.62) | 4.39 (0.77, 8.49) p=0.04000 | 0.42 (0.25, 0.61) p=0.00000 | 0.76 (0.55, 1.07) p=0.10000 | 1.06 (0.76, 1.62) p=0.86000 | 1.58 (1.08, 2.34) p=0.02000 |
|  | JAVELIN^b^ | AVE+AXI | 44.9 months | 26.57 (24.35, 28.74) | 18.38 (17.00, 19.47) | 8.20 (5.55, 10.39) p=0.00000 | 0.51 (0.37, 0.64) p=0.00000 | 0.61 (0.48, 0.82) p=0.00000 | 0.65 (0.49, 0.87) p=0.00000 | 0.58 (0.44, 0.75) p=0.00000 |
|  | KEYNOTE-426^c^ | PEM+AXI | 70.9 months | 29.33 (25.34, 32.82) | 23.65 (21.55, 25.90) | 5.68 (1.18, 9.78) p=0.04000 | 0.59 (0.49, 0.76) p=0.00000 | 0.74 (0.59, 0.96) p=0.02000 | 0.84 (0.68, 1.10) p=0.18000 | 0.89 (0.69, 1.13) p=0.40000 |
|  | CheckMate 9ER^d^ | NIVO+CABO | 39.3 months | 24.01 (20.69, 28.00) | 19.22 (17.61, 20.53) | 4.79 (0.99, 9.09) p=0.02000 | 0.58 (0.38, 0.89) p=0.04000 | 0.72 (0.49, 1.07) p=0.12000 | 0.86 (0.60, 1.24) p=0.44000 | 0.72 (0.49, 1.04) p=0.16000 |
| **Log-logistic all covariates** | CheckMate 214^a^ | NIVO+IPI | 70.3 months | 28.98 (22.66, 35.33) | 24.64 (22.62, 26.62) | 4.35 (-3.25, 11.65) p=0.22000 | 0.42 (0.24, 0.75) p=0.00000 | 0.74 (0.48, 1.21) p=0.16000 | 1.04 (0.68, 1.56) p=0.90000 | 1.61 (1.02, 2.51) p=0.06000 |
|  | JAVELIN^b^ | AVE+AXI | 44.9 months | 26.25 (21.06, 31.19) | 18.38 (17.00, 19.47) | 7.87 (2.27, 13.18) p=0.00000 | 0.55 (0.31, 0.86) p=0.00000 | 0.66 (0.40, 0.94) p=0.04000 | 0.69 (0.43, 0.97) p=0.00000 | 0.60 (0.39, 0.87) p=0.00000 |
|  | KEYNOTE-426^c^ | PEM+AXI | 70.9 months | 28.70 (20.94, 35.90) | 23.65 (21.55, 25.90) | 5.06 (-2.25, 11.51) p=0.16000 | 0.57 (0.36, 0.97) p=0.06000 | 0.72 (0.48, 1.19) p=0.22000 | 0.84 (0.55, 1.28) p=0.40000 | 0.91 (0.63, 1.31) p=0.58000 |
|  | CheckMate 9ER^d^ | NIVO+CABO | 39.3 months | 23.799 (19.841, 29.557) | 19.22 (17.61, 20.53) | 4.582 (0.0544, 10.450) p=0.06000 | 0.60 (0.29, 1.06) p=0.12000 | 0.75 (0.41, 1.23) p=0.28000 | 0.87 (0.52, 1.51) p=0.66000 | 0.71 (0.48, 1.20) p=0.36000 |
| **Log-logistic no covariates** | CheckMate 214^a^ | NIVO+IPI | 70.3 months | 27.640 (24.805, 29.729) | 24.64 (22.62, 26.62) | 3.004 (0.0516, 6.091) p=0.04000 | 0.53 (0.39, 0.66) p=0.00000 | 0.81 (0.68, 1.09) p=0.16000 | 1.10 (0.89, 1.43) p=0.48000 | 1.65 (1.27, 2.26) p=0.00000 |
|  | JAVELIN^b^ | AVE+AXI | 44.9 months | 25.07 (23.22, 26.69) | 18.38 (17.00, 19.47) | 6.69 (4.46, 8.65) p=0.00000 | 0.62 (0.49, 0.80) p=0.00000 | 0.69 (0.59, 0.91) p=0.00000 | 0.70 (0.58, 0.92) p=0.02000 | 0.62 (0.50, 0.83) p=0.00000 |
|  | KEYNOTE-426^c^ | PEM+AXI | 70.9 months | 27.64 (25.71, 29.28) | 23.65 (21.55, 25.90) | 3.99 (1.23, 6.55) p=0.02000 | 0.73 (0.58, 0.94) p=0.02000 | 0.85 (0.67, 1.06) p=0.16000 | 0.92 (0.73, 1.16) p=0.54000 | 0.94 (0.74, 1.22) p=0.58000 |
|  | CheckMate 9ER^d^ | NIVO+CABO | 39.3 months | 23.34 (21.86, 25.03) | 19.22 (17.61, 20.53) | 4.12 (2.00, 6.51) p=0.00000 | 0.66 (0.50, 0.89) p=0.00000 | 0.79 (0.62, 1.03) p=0.08000 | 0.86 (0.66, 1.16) p=0.40000 | 0.70 (0.51, 1.07) p=0.12000 |
| **1 knot spline hazard** | CheckMate 214^a^ | NIVO+IPI | 70.3 months | 28.19 (24.18, 32.66) | 24.64 (22.62, 26.62) | 3.55 (-0.82, 7.96) p=0.10000 | 0.46 (0.30, 0.61) p=0.00000 | 0.74 (0.48, 1.00) p=0.06000 | 1.01 (0.70, 1.56) p=0.90000 | 1.59 (1.10, 2.56) p=0.04000 |
|  | JAVELIN^b^ | AVE+AXI | 44.9 months | 26.74 (24.56, 28.95) | 18.38 (17.00, 19.47) | 8.36 (6.05, 10.76) p=0.00000 | 0.53 (0.40, 0.68) p=0.00000 | 0.61 (0.46, 0.75) p=0.00000 | 0.62 (0.46, 0.80) p=0.00000 | 0.55 (0.43, 0.74) p=0.00000 |
|  | KEYNOTE-426^c^ | PEM+AXI | 70.9 months | 29.32 (26.433, 31.78) | 23.65 (21.55, 25.90) | 5.67 (1.14, 8.55) p=0.00000 | 0.61 (0.46, 0.87) p=0.00000 | 0.72 (0.57, 0.92) p=0.02000 | 0.79 (0.63, 0.97) p=0.04000 | 0.86 (0.63, 1.14) p=0.30000 |
|  | CheckMate 9ER^d^ | NIVO+CABO | 39.3 months | 22.64 (18.47, 27.03) | 19.22 (17.61, 20.53) | 3.42 (-1.22, 8.03) p=0.16000 | 0.73 (0.40, 1.09) p=0.16000 | 0.83 (0.53, 1.23) p=0.48000 | 0.95 (0.61, 1.71) p=0.82000 | 0.86 (0.48, 1.80) p=0.66000 |
| **1-knot spline normal** | CheckMate 214^a^ | NIVO+IPI | 70.3 months | 29.70 (25.96, 32.83) | 24.64 (22.62, 26.62) | 5.06 (0.83, 8.79) p=0.021978 | 0.43 (0.26, 0.58) p=0.00000 | 0.70 (0.50, 1.05) p=0.10000 | 1.03 (0.76, 1.51) p=0.96000 | 1.58 (1.14, 2.52) p=0.02000 |
|  | JAVELIN^b^ | AVE+AXI | 44.9 months | 26.640 (24.805, 28.346) | 18.38 (17.00, 19.47) | 8.26 (5.89, 10.41) p=0.00000 | 0.50 (0.38, 0.70) p=0.00000 | 0.58 (0.46, 0.78) p=0.00000 | 0.62 (0.48, 0.82) p=0.00000 | 0.56 (0.43, 0.73) p=0.00000 |
|  | KEYNOTE-426^c^ | PEM+AXI | 70.9 months | 29.727 (26.673, 32.247) | 23.65 (21.55, 25.90) | 6.08 (2.41, 9.15) p=0.00000 | 0.60 (0.41, 0.85) p=0.00000 | 0.73 (0.55, 0.97) p=0.04000 | 0.80 (0.62, 1.05) p=0.10000 | 0.88 (0.68, 1.13) p=0.38000 |
|  | CheckMate 9ER^d^ | NIVO+CABO | 39.3 months | 24.90 (21.40, 28.76) | 19.22 (17.61, 20.53) | 5.68 (2.30, 9.93) p=0.00000 | 0.58 (0.32, 1.05) p=0.08000 | 0.69 (0.50, 0.97) p=0.04000 | 0.79 (0.55, 1.12) p=0.30000 | 0.67 (0.45, 1.09) p=0.12000 |
| **Exponential** | CheckMate 214^a^ | NIVO+IPI | 70.3 months | 28.17 (24.57, 32.66) | 24.64 (22.62, 26.62) | 3.53 (-0.63, 8.47) p=0.14000 | 0.45 (0.29, 0.56) p=0.00000 | 0.69 (0.48, 0.92) p=0.02000 | 0.96 (0.66, 1.41) p=0.82000 | 1.53 (1.03, 2.32) p=0.04000 |
|  | JAVELIN^b^ | AVE+AXI | 44.9 months | 26.68 (24.46, 28.64) | 18.38 (17.00, 19.47) | 8.31 (5.73, 10.65) p=0.00000 | 0.50 (0.40, 0.60) p=0.00000 | 0.53 (0.42, 0.66) p=0.00000 | 0.57 (0.46, 0.75) p=0.00000 | 0.54 (0.42, 0.69) p=0.00000 |
|  | KEYNOTE-426^c^ | PEM+AXI | 70.9 months | 29.32 (26.79, 32.74) | 23.65 (21.55, 25.90) | 5.67 (1.86, 9.55) p=0.00000 | 0.60 (0.46, 0.75) p=0.00000 | 0.66 (0.51, 0.82) p=0.00000 | 0.76 (0.57, 0.93) p=0.04000 | 0.86 (0.63, 1.11) p=0.16000 |
|  | CheckMate 9ER^d^ | NIVO+CABO | 39.3 months | 22.52 (18.53, 27.06) | 19.22 (17.61, 20.53) | 3.30 (-0.21, 8.37) p=0.12000 | 0.66 (0.43, 1.11) p=0.12000 | 0.74 (0.51, 1.20) p=0.26000 | 0.87 (0.57, 1.40) p=0.64000 | 0.80 (0.52, 1.27) p=0.50000 |
| **KM only** | CheckMate 214^a^ | NIVO+IPI | 70.3 months | 28.51 (26.35, 31.16) | 24.64 (22.62, 26.62) | 3.88 (0.95, 6.80) p=1.00000 | 0.93 (0.68, 1.28) p=0.66684 | 0.61 (0.48, 0.78) p=3.3944e-05 | 0.63 (0.51, 0.78) p=8.8879e-06 | 0.64 (0.53, 0.78) p=5.1791e-06 |
|  | JAVELIN^b^ | AVE+AXI | 44.9 months | 25.43 (23.67, 27.19) | 18.38 (17.00, 19.47) | 7.06 (4.90, 9.23) p=1.00000 | 0.93 (0.66, 1.31) p=0.69000 | 0.66 (0.52, 0.85) p=0.00090022 | 0.65 (0.52, 0.80) p=5.0656e-05 | 0.65 (0.53, 0.80) p=1.7483e-05 |
|  | KEYNOTE-426^c^ | PEM+AXI | 70.9 months | 28.51 (26.14, 30.50) | 23.65 (21.55, 25.90) | 4.87 (1.86, 7.76) p=1.00000 | 0.91 (0.64, 1.27) p=0.56319 | 0.63 (0.49, 0.81) p=0.00030829 | 0.69 (0.55, 0.86) p=0.00069869 | 0.74 (0.61, 0.91) p=0.0038137 |
|  | CheckMate 9ER^d^ | NIVO+CABO | 39.3 months | 23.672 (22.313, 25.193) | 19.22 (17.61, 20.53) | 4.46 (2.79, 6.88) p=1.00000 | 0.91 (0.63, 1.33) p=0.62744 | 0.68 (0.52, 0.90) p=0.0055019 | 0.67 (0.53, 0.85) p=0.00071778 | 0.66 (0.53, 0.82) p=0.00013588 |

* RMST is measured in months up to 57.82 months CLEAR follow-up, or the follow-up of the comparator, whichever is shortest. P-values are one-sided.

a Adjusted for IMDCP_FAVORABLE, PDL1_L1, ORGSGR1_GE2, LBONEN, LLYMPHN, LLIVEN

b Adjusted for MSKCCP_FAVORABLE, IMDCP_FAVORABLE, ORGSGR1_GE2

c Adjusted for MSKCCP_FAVORABLE, IMDCP_FAVORABLE, PDL1_L1, ORGSGR1_GE2, LBONEN, LLYMPHN, LLIVEN

d Adjusted for IMDCP_FAVORABLE, PDL1_L1, ORGSGR1_GE2, LBONEN, LLYMPHN, LLIVEN

AVE+AXI=avelumab + axitinib; HR=hazard ratio; KM=Kaplan-Meier; LEN+PEM=lenvatinib + pembrolizumab; NIVO+CABO=nivolumab + cabozantinib; PEM+AXI=pembrolizumab + axitinib; PFS=progression-free survival; RMST=Restricted Mean Survival Time
